# Design and descriptive epidemiology of the Infectious Diseases of East African Livestock (IDEAL) project, a longitudinal calf cohort study in western Kenya

**DOI:** 10.1186/1746-6148-9-171

**Published:** 2013-08-30

**Authors:** Barend Mark de Clare Bronsvoort, Samuel Mwangi Thumbi, Elizabeth Jane Poole, Henry Kiara, Olga Tosas Auguet, Ian Graham Handel, Amy Jennings, Ilana Conradie, Mary Ndila Mbole-Kariuki, Philip G Toye, Olivier Hanotte, JAW Coetzer, Mark EJ Woolhouse

**Affiliations:** 1 The Roslin Institute and Royal (Dick) School of Veterinary Studies, University of Edinburgh, Roslin, UK; 2 ILRI, P.O. Box 30709, Nairobi 00100, Kenya; 3 Centre for Immunology, Infection & Evolution, University of Edinburgh, Kings Buildings, Edinburgh, UK; 4 University of Nottingham, University Park, Nottingham, NG7 2RD, UK; 5 Department of Veterinary Tropical Diseases, Faculty of Veterinary Science, University of Pretoria, Private bag X04, Onderstepoort, South Africa; 6 Centre for Clinical Infection and Diagnostics Research, Department of Infectious Diseases, King’s College London and Guy’s and St Thomas’ NHS Foundation Trust, London, UK

**Keywords:** Cattle, Infectious disease, Kenya, Longitudinal study, Cohort, Epidemiology, Study design

## Abstract

**Background:**

There is a widely recognised lack of baseline epidemiological data on the dynamics and impacts of infectious cattle diseases in east Africa. The Infectious Diseases of East African Livestock (IDEAL) project is an epidemiological study of cattle health in western Kenya with the aim of providing baseline epidemiological data, investigating the impact of different infections on key responses such as growth, mortality and morbidity, the additive and/or multiplicative effects of co-infections, and the influence of management and genetic factors.

A longitudinal cohort study of newborn calves was conducted in western Kenya between 2007-2009. Calves were randomly selected from all those reported in a 2 stage clustered sampling strategy. Calves were recruited between 3 and 7 days old. A team of veterinarians and animal health assistants carried out 5-weekly, clinical and postmortem visits. Blood and tissue samples were collected in association with all visits and screened using a range of laboratory based diagnostic methods for over 100 different pathogens or infectious exposures.

**Results:**

The study followed the 548 calves over the first 51 weeks of life or until death and when they were reported clinically ill. The cohort experienced a high all cause mortality rate of 16% with at least 13% of these due to infectious diseases. Only 307 (6%) of routine visits were classified as clinical episodes, with a further 216 reported by farmers. 54% of calves reached one year without a reported clinical episode. Mortality was mainly to east coast fever, haemonchosis, and heartwater. Over 50 pathogens were detected in this population with exposure to a further 6 viruses and bacteria.

**Conclusion:**

The IDEAL study has demonstrated that it is possible to mount population based longitudinal animal studies. The results quantify for the first time in an animal population the high diversity of pathogens a population may have to deal with and the levels of co-infections with key pathogens such as *Theileria parva*. This study highlights the need to develop new systems based approaches to study pathogens in their natural settings to understand the impacts of co-infections on clinical outcomes and to develop new evidence based interventions that are relevant.

## Background

It is estimated that by 2050 the global population will have risen to 9 billion with much of this growth predicted to occur in sub-saharan Africa [1]. There is therefore an urgent need to improve food production in these regions, and livestock production constitutes an essential part of this. In addition to providing food through milk and meat, livestock also provide hides, draught power, manure for fertiliser, building and fuel, capital reserves and cultural services and in many marginal regions are the only useful way of utilising poor quality grazing land. Livestock are key to poor peoples’ livelihoods and offer an important route out of poverty.

Constraints on livestock production are varied and include nutrition, management, access to markets, natural catastrophes and importantly infectious disease (eg. [2]). Sub-Saharan Africa (SSA) harbours 12 of the 15 former World Organisation for Animal Health (O.I.E.) list A diseases considered most contagious including African swine fever, Rift Valley fever and African horse sickness. In addition, many less contagious but arguably more important diseases such as East Coast fever, trypanosomosis, brucellosis and leptospirosis are widespread. A systematic literature review on causes of morbidity and mortality among smallholder dairy farms in Eastern and South Africa identified tick-borne diseases, diarrhoea and trypanosomiasis as the most commonly documented causes of mortality [3]. This limits production directly but also ensures these regions are unable to trade animals and their products in international markets [4]. However, rinderpest is a clear example where a regional approach has produced a highly successful eradication programme and the world is now rinderpest free [5]. This points to the need for targeted research to understand the full spectrum of disease problems in a farming system and how an integrated control package might release the genetic potential of the existing livestock while maintaining genetic resilience to environmental or emerging disease threats.

Previous work in infectious disease epidemiology has focused on single disease studies eg. Zhang [6], Bronsvoort [7] and Gachohi [8] or a few closely related diseases eg. [9] but, in reality, organisms are normally infected with a number of more or less pathogenic organisms at any one time. There is increasing scientific interest in how pathogens interact, within both individuals [10,11] and populations [12]. Examples include studies of viruses, bacteria, protozoa and helminth infections in both humans and livestock [11-20]. These interactions can be positive or negative and involve mechanisms such as: common risk factors and transmission routes (including shared vectors); non-specific immune responses; cross-reactive acquired immune responses; increased susceptibility of immuno-suppressed or immuno-compromised hosts; non-specific effects of genetic polymorphisms and nutritional deficiencies; the demographic and behavioural impacts of infectious diseases and of intervention measures. There may also be consequences of variations in the timing and ordering of exposure, infection, and disease caused by different pathogens, including responses to vaccinations [21,22].

Animal health research in SSA has traditionally focussed on specific infections, particularly tick-borne and tsetse-borne diseases, not necessarily because they are the major diseases of cattle kept by the poor in these environments, but because they are known historically to be serious constraints to commercial systems using improved breeds. However, livestock in the tropics are routinely exposed to a wide variety of pathogens [23] whose direct and indirect impacts on animal health are unlikely to be independent of one another. Local breeds have been reared in these heavy disease challenge settings for many centuries which has resulted in selection for broad disease resistance likely at the expense of higher production [24]. Yet there have been no integrated studies of the co-distribution, co-incidence and overall impact of the major infectious diseases of livestock in the tropics. There is a need for detailed knowledge of the burden of infectious diseases impose on livestock as a prerequisite to informed decision making, resource allocation, prioritisation of research and selection of interventions. However, there is growing evidence that disease impacts cannot be fully understood by reference to single infections in isolation [25]. Instead, a holistic approach is required which considers both direct and indirect interactions between pathogens and the effects of these on the epidemiologies of infectious diseases of cattle and of the disease burdens they impose and, ultimately, of their impacts on human welfare [16,26].

The Infectious Diseases of East African Livestock (IDEAL) project is a multi-disciplinary study which addresses two major issues: 1) the widely recognised lack of baseline epidemiological data on the dynamics and impacts of infectious diseases of cattle in the tropics; and 2) improving understanding of interactions between multiple infections and their sequelae by testing two specific hypotheses: i) that the negative impacts of different infections are not independent; ii) that ‘positive’ traits (e.g. resistance to infection, higher growth rates, low morbidity) cluster in certain individuals. In order to test these hypotheses we designed a longitudinal epidemiological field study to follow a random sample of newborn indigenous short horn zebu calves, with known genotype, through the first 12 months of life and to monitor them closely to identify when and what pathogens they were exposed to and the impact these had individually and in combination.

This paper describes the study design and reports the descriptive epidemiology of the IDEAL project. In particular we provide baseline data on the farm demographics and characterise the small holder African Shorthorn Zebu farming system of western Kenya which may be representative of the wider Lake Victoria basin. We also report the overall infectious disease related mortality rates and incidence of clinical episodes, the range of pathogens and exposures observed and the proportion of the cohort affected by each to provide a context for future papers on specific aspects of mortality and morbidity.

## Methods

### Study setting

There has been intensive work to define the distribution of different agricultural production systems in East Africa (eg. [27,28]). This study focused on a specific production system, sedentary mixed crop-livestock small holdings. This system encompasses >50% of poor people (defined as income below US<DOLLAR/>15 per month [29]) resident in East Africa [30], covers extensive areas of Kenya and beyond, and is of increasing importance as populations grow.

The study site was an area of western Kenya approximately 45 x 90km covering some or all of Busia (95.9%), Teso (96.3%), Siaya (55.5%), Butere/Mumias (26.9%) and Bungoma (20.4%) districts. Each district is further divided into sub-locations which are the smallest administrative unit in Kenya for which data was available on cattle numbers. A SL typically contains 60 to 90 households per km^2^ and is 10-20km^2^ in area. Land plots are typically 1-5 ha in size, with around 60% of households owning 2-3 breeding cattle grazed communally. The study site included 280 sub-locations (excluding 2 that were in Busia and Mumias towns) across 5 agro-ecological zones (AEZ). AEZ is a way to describe the type of land and its suitability for different crops and combines data on soil, topography, and climate. The areas of Kakamega, Vihiga, Lugari and Mt Elgon districts were not included as they were considered less representative of smallholder livestock farmers in East Africa (e.g. Mt Elgon slope, large-scale dairy farming more prominent) and due to logistic restrictions (i.e. the diagnostic laboratory was in Busia town, to which samples were transported daily).

### Study design and recruitment

**Figure 1:**
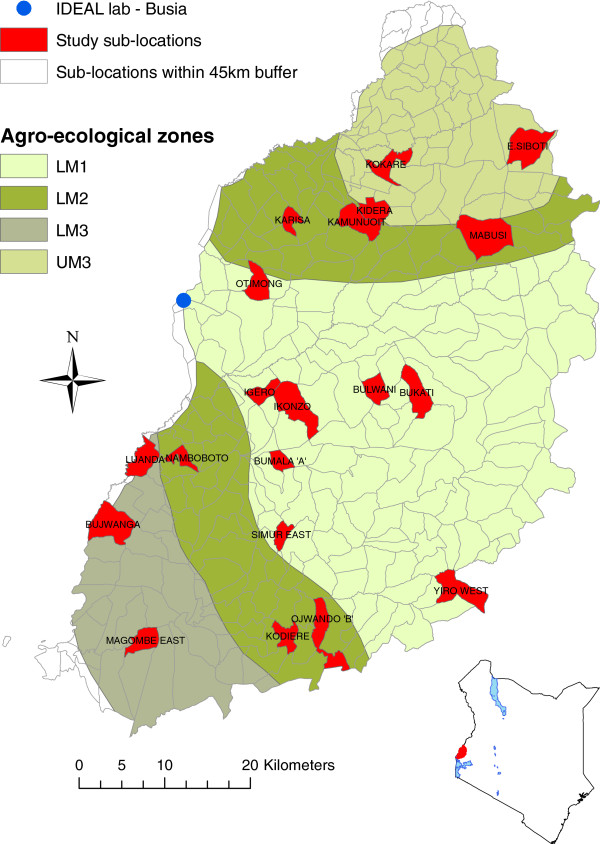
Map of western Kenya showing the study area, agro-ecological zones and sub-locations (selected sub-locations highlighted).

A stratified 2-stage random cluster sample of calves was drawn. The 1st stage cluster sample (by sub-location) was selected by random sampling sub-locations with replacement within each AEZ stratum. A total of 20 sub-locations were selected (Figure 1 and Table 1). A second stage sample size of 28 calves per sub-location was chosen at random to achieve the desired minimum sample size of 500 calves (based on logistical constraints and ability to detect a minimum relative risk of 3 with 80% power) and to allow for some losses (Table 2). A reporting system was established in each of the 20 selected sub-locations using a reporting pathway from Farmer → Sub-location-chief → Sub-chief → IDEAL Office. Each recruitment day the animal heath assistants (AHA) collated the eligible calf births for the sub-location and randomly selected 1-3 calves randomly from a hat each day. In order to be eligible the calf had to meet a set of specific selection criteria which were (1) the calf had to be between 3 and 7 days old at recruitment; (2) it was not as a result of artificial insemination; and (3) the dam was not managed under zero-grazing conditions. These criteria were set to give a reasonable window to capture calves being born without being too old and to avoid recruitment of exotic breeds rather than indigenous cattle. The sub-locations were visited on a rolling 5 week cycle to ensure there was an even distribution of calves across space and season. Calves were recruited over the 5 week cycle with 4/20 sub-locations being visited each week, taking 2 years to recruit the complete cohort. Only one calf per dam was recruited and a farmer could only have one calf at a time in the study. Recruitment was conditional on the farmer allowing access to the calf and willingness to report clinical episodes to the project and not “self treat". A flat rate of compensation was agreed with the local veterinary office for this. Owners were asked to call the IDEAL team if a calf was observed to be ill between visits and one of the project veterinary surgeons would examine the calf and treat if considered to be seriously ill or a welfare issue. Calves were censored after any visit where a treatment was begun.

**Table 1:** Selected sub-locations with census/demographic characteristics (taken from the Human Population Census in Kenya 1999)

**AEZ**	**Sub-location**	**No. households**	**Area(km**^**2**^**)**	**Cattle density per km**^**2**^	**Average herd size**
UM3	East Siboti	1245	15.80	2439	3.4
	Kokare	325	8.29	937	6.1
	Kidera	314	7.36	728	4.8
LM1	Yiro West	1361	13.70	1187	3.9
	Simur East	415	4.32	425	3.8
	Igero	532	5.60	681	3.6
	Bumala A	724	4.38	222	2.3
	Ikonzo	1421	16.40	598	2.8
	Bulwani	478	6.87	578	3.2
	Bukati	993	11.20	1259	2.5
	Otimong	506	8.66	869	4.1
LM2 middle	Mabusi	1575	22.50	1575	3.1
	Kamunuoit	556	11.00	957	4.0
	Karisa	292	4.63	247	2.2
LM2 South	Ojwando B	832	12.60	1095	4.6
	Kodiere	630	6.38	849	4.7
	Namboboto	351	4.46	220	2.7
LM3	Luanda	726	9.76	730	4.7
	Bujwanga	1025	16.70	792	4.2
	Magombe East	578	7.67	852	5.4

**Table 2:** Distribution of sub-locations (SL) across agroecological zones (AEZs) in western Kenya and number selected for the IDEAL study

**AEZ**	**No. SL/AEZ**	**Proportion/AEZ**	**No. SLs selected**
LM1	114	0.40	8
LM2	86	0.30	6
LM3	28	0.10	3
LM4	4	0.01	0
UM3	53	0.19	3
Total	285		20

**Figure 2:**
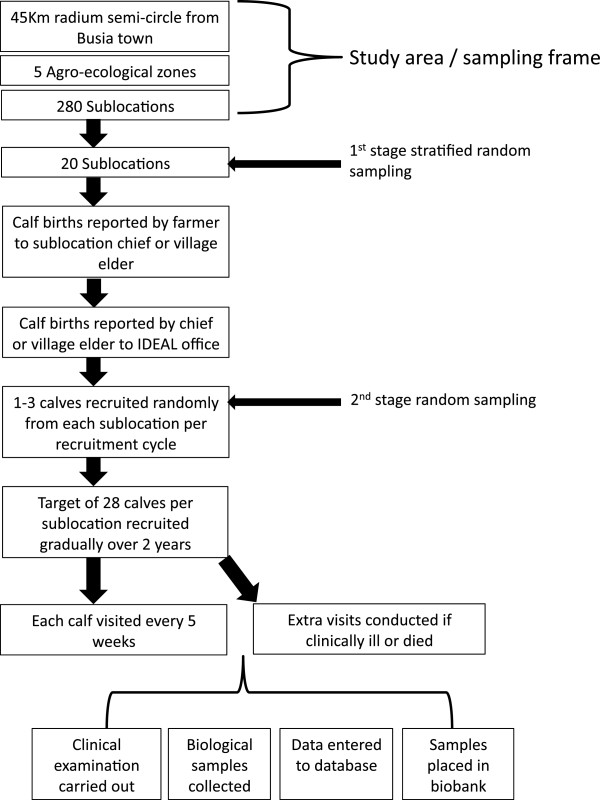
Schema showing the design and sampling used in the IDEAL project.

Upon recruitment a household questionnaire was completed by interview with the owner/head of the household. The questionnaire included questions about the farm size, crops, water sources, and other livestock. The dam was examined and a form completed and if it or the calf failed any of the eligibility criteria, the calf was excluded. The calf was then examined and a recruitment form and routine visit form completed. The calf was examined for congenital deformities and excluded if any were found. This is summarised in Figure 2.

#### Data collection and training of data collectors

Data collection took place at the farm. A team comprising a veterinary surgeon/senior AHA and two AHAs went to each animal and followed a standard protocol for the physical examination and collection of compulsory samples. If the dam was also being visited there was an additional protocol for dam examinations. The AHAs were also trained in data collection and all questionnaires and data collection tools were piloted over about 9 months during the set-up phase of the project in western Kenya. Data were collected via a hand held Palm OS®; Personal-Digital Assistant (PDAs) and simultaneously on a paper questionnaire form. Barcodes were used to identify and link samples to individual animals. At the diagnostic field laboratory in Busia, data were downloaded from the hand held device to a database and cross-checked against the paper records and any discrepancies resolved with the AHA who collected the data.

#### Routine clinical examination of calves

The clinical examination consisted of a systematic physical examination of the calf. This included observation of the animal at rest, posture, alertness, rectal temperature, weight, girth, FAMACHA score [31], mucus membrane colour, skin elasticity, presence and species of ticks and other ectoparasites and full palpation of the body checking for lesions and discharges. In addition to the physical examination of the calf a short questionnaire was used to update other activities on the farm such as any animal purchases or sales, treatment of the other livestock or cases of illness in other livestock.

**Figure 3:**
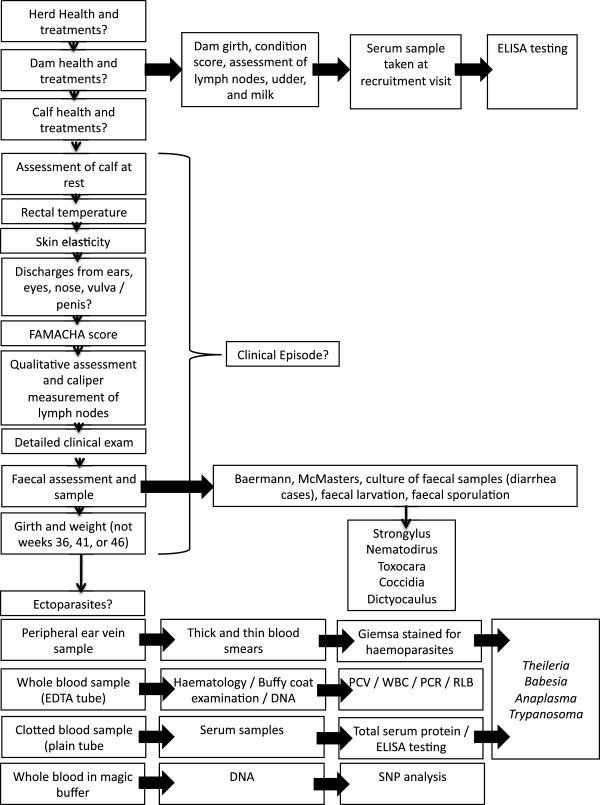
Schema showing the types and timings of clinical examination of calves and the types of sample collection for the IDEAL project.

A standard set of samples were collected at recruitment (7D), 5 weekly (5W), and 51 weeks (Y) visits as detailed below. A marginal ear vein sample was used to make a thick and a thin blood smear to screen for haemoparasites and for manual differential cell counts following shipment to Pretoria University. A jugular vein sample was collected into plain tubes for total serum protein estimation using a refractometer (model RHC-200ATC, Westover Scientific) and storage for antibody screening for a range of haemoparasites, bacteria and viruses and 0.5ml was added to RNAlater®; (Ambion®;) and stored at 4°C. An EDTA sample with ‘magic buffer‘ was collected for genomic analysis (7D only). An EDTA sample for: (a) DNA extraction for pathogens; (b) direct microscopy on thick and thin smears for haemoparasities and (d) routine haematology including WBC, RBC, PCV, MCV, HGB, MCH, MCHC using a Sysmex pocH-100iV Diff automated blood analyser (Sysmex®; Europe GMBH) was also collected. A further EDTA sample was stored at -80°C until DNA extraction and shipping to Pretoria University for screening (Y or last visit before death) for a large range of blood borne parasites using the reverse line blot (RLB) [32]. A heparinised blood sample was collected for *Mycobacterium bovis* screening using the “Bovigam” ELISA (Prionics®;, Celtic Diagnostics Ltd., Ireland) (Y only). In addition samples were collected for white blood cell stimulation, however, this was discontinued early in the study because of logistical constraints. Faecal samples were collected via rectal palpation for screening for helminths using standard techniques [33]. Samples were divided and one part put in a plastic bag and stored overnight at 4°C for screening by McMasters technique for strongyle eggs, by the direct Baermans technique for *Dictyocaulus vivperia* larvae, by Ziehl-Neelsen stained smear for *Cryptosporidium spp.* and *M. avium paratuberculosis* and by sedimentation for fluke species eggs. The second part was stored in a pot at room temperature overnight and then prepared for larval culture to speciate strongle eggs. Samples with >2000 coccidia oocyts were also cultured to type the species of coccidia present. Three superficial skin snips were taken from the ventral abdomen and incubated directly in RPMI-1640 (Sigma-Aldrich®;) to screen for *Onchocerca spp.* microfilaria [34]. Results from diagnostic tests done in the field laboratory in Busia were entered directly in a separate laboratory database. In addition at the final visit to a calf a standard set of measurements of height at wither, nose to tail length and phenotypical measures such as coat colour and hump and dewlap were recorded. This is summarised in Figure 3.

#### Clinical episodes and post mortem examinations in calves

In addition to routine clinical examinations and in order to capture as many clinical episodes as possible local AHAs working for the Kenyan Department of Veterinary Services in the sub-locations made weekly visits to each calf. These weekly visits involved a limited clinical examination focusing on identifying any acute disease and in particular any pyrexic or traumatic episodes. In the event that they identified pyrexia, enlarged lymph nodes or respiratory distress, they contacted an IDEAL project veterinary surgeon and an extra non-routine visit was made. The main triggers for a visit were a temperature of >40.5°C, generalised lymphadenopathy, anorexia, diarrhoea, generalised skin conditions, non-weight bearing lameness, coughing or respiratory distress. However each case report was considered and was visited depending on history and if there was believed to be a compromise in welfare. A full clinical examination was carried out and additional samples were collected based on the clinical syndrome observed. These included swabs of any discharges for bacteriological culture and typing, viral swabs and heparin blood samples for virological culture, and needle aspirates from enlarged lymph nodes for microscopy. If calves were in a severely diseased state the project veterinarian used their professional judgement and a set of criteria agreed with the ethics committee at University of Edinburgh/International Livestock Research Institute and the animal was euthanised if necessary.

In the event that an animal died or was euthanised a full gross post mortem examination was carried out following standard veterinary approaches working through the body systems. A standard set of tissues was collected from each animal, including lung, liver, duodenum, ileum and lymph nodes, with additional samples specific to the suspected aetiology where appropriate. In the event of a history of sudden death a marginal ear vein blood smear was made and stained with methylene blue and checked for the presence of anthrax bacilli prior to further examination. In the event of a positive smear no post mortem was performed and the carcass buried. If there were neurological signs and/or a history consistent with rabies the head was removed and sent for testing at the Central Veterinary Laboratories at Kabete, Kenya and the remainder of the carcass incinerated. For those animals with neurological signs and no history of possible bites, a brain smear was prepared using the standard approach for identification of *E. ruminantium* the cause of heartwater disease.

### Examination of the dams

In addition to the above the calf’s dam was examined at each visit. At recruitment a full clinical examination was done (including manual palpation of the udder for evidence of mastitis), the girth measured and the animal was condition scored using a standard 10 point score [35]. In addition phenotypic measurements of coat colour and pattern, horn length and shape, ear shape, size of hump and dewlap were recorded. Two plain and 3 EDTA vacuutainers of blood were collected for possible use later. At each 5 weekly visit up to the visit after the calf was weaned the dam was re-examined, the girth was measured, the animal was condition scored and the udder examined.

In the initial phase of the study we attempted to collect milk samples from dams at each visit. These are low production animals and have very small udders and teats compared to a holstein for example. In the majority of cases we were unable to collect samples as the calf would have suckled before we arrived and/or the owner had milked the dam. Similarly the AHAs were initially trained to use the California milk test [36] but again it proved very difficult to get enough milk to test. Both these activities were suspended after the first 3 months in December 2007.

### Laboratory analysis

A full list of pathogens that the project attempted to identify that we believed likely to be present in this setting is given in Table 3 and includes 100 different pathogens. The various techniques used and the time points at which they were done are also provided for reference. In some cases there is overlap as some techniques will only differentiate to genus level while others will allow species specific identification.

**Table 3:** Pathogens screened for during the study

**Pathogen**	**Test**	**Visits tested**	**Pathogen**	**Test**	**Visits tested**
*Actinomyces sp.*	RB	CE	*Hepatozoon spp.* catch-all	RLB	Y
*Actinomycetes*	RB	CE	*Hyalomma spp.*	CL	7D, 5W, Y
*Amblyomma variegatum*	CL	7D, 5W, Y	*Hypoderma bovis*	CL	7D, 5W, Y
*Anaplasma bovis*	RLB	Y	*Klebsiella ozaenae*	RB	CE
*Anaplasma centrale*	RLB	Y	*Klebsiella pneumoniae*	RB	CE
*Anaplasma marginale*	RLB	Y	*Listeria spp.*	RB	CE
*Anaplasma ovis*	RLB	Y			
*Anaplasma phagocytophilum*	RLB	Y	Lumpy skin disease	PCR	CE
*Arcanobacterium pyogenes*	RB	CE	*Micrococcus spp.*	RB	CE
*Babesia bicornis*	RLB	Y	*Moniezia spp.*	FM,FC	7D, 5W, Y
*Babesia bigemina*	RLB	Y	*M. avium paratuberculosis*	ZN	Y
*Babesia bovis*	RLB	Y	*Nematodirus spp.*	FM,FC	7D, 5W, Y
*Babesia caballi*	RLB	Y	*Non-pathogenic Staphylococci*	RB	CE
*Babesia canis*	RLB	Y	*Oesophagostomum radratium*	FM,FC	7D, 5W, Y
*Babesia divergens*	RLB	Y	*Onchocerca spp.*	SNP,MIC	Y
*Babesia felis*	RLB	Y	*Ostertagia ostertagi*	FM+FC	7D, 5W, Y
*Babesia gibsoni* Japan	RLB	Y	*Pasteurella multocida*	RB	CE
*Babesia microti*	RLB	Y	*Rickettsia spp.* catch-all	RLB	Y
*Babesia motasi*	RLB	Y	*Rickettsia spp. (DnS14) raoultii*	RLB	Y
*Babesia odocoilei*	RLB	Y	*Riphicephalus appendiculatus*	CL	7D, 5W, Y
*Babesia ovis*	RLB	Y	Rotavirus	ELISA	CE
*Babesia rossi*	RLB	Y	*Salmonella spp.*	RB	CE
*Babesia vogeli*	RLB	Y	*Sarcocystis spp.*	HIS	PM
*Bacillus anthracis*	RB	PM	*Staphylococcus aureus*	RB	CE
Bluetongue virus	PCR	Y, CE	*Staphylococcus epidermicus*	RB	CE
*Bacillus spp.*	RB	CE	*Staphylococcus epidermidis*	RB	CE
*Boophilus spp.*	CL	7D, 5W, Y	*Staphylococcus spp.*	RB	CE
*Borrelia afzelii*	RLB	Y	*Streptococcus bovis*	RB	CE
*Borrelia burgdorferi s. lato*	RLB	Y	*Streptococcus spp.*	RB	CE
*Borrelia burgdorferi s. stricto*	RLB	Y	*Theileria annae*	RLB	Y
*Borrelia garinii*	RLB	Y	*Theileria annulata*	RLB	Y
*Borrelia valaisiana*	RLB	Y	*Theileria bicornis*	RLB	Y
*Bunostomum trigonocephalum*	FM	7D, 5W, Y	*Theileria buffeli*	RLB	Y
Bovine Viral Diarrrhoea Virus	ELISA - ag	Y	*Theileria cervi*	RLB	Y
*Calicophoron spp.*	FM,FC	7D, 5W, Y	*Theileria equi*	RLB	Y
*Chabertia ovina*	FM,FC	7D, 5W, Y	*Theileria equi*-like	RLB	Y
*Clostridium spp.*	RB	CE	*Theileria lestoquardi*	RLB	Y
*Coccidia spp.*	FM,FC	7D, 5W, Y	*Theileria mutans*	RLB	Y
*Coccobacillary*	RB	CE	*Theileria orientalis* 1	RLB	Y
*Cooperia spp.*	FM,FC	7D, 5W, Y	*Theileria parva*	RLB,PCR	Y
*Corynebacterium spp.*	RB	CE	*Theileria spp.* (buffalo)	RLB	Y
*Cryptosporidium spp.*	ZN,MIC	7D, 5W, Y	*Theileria spp.* (duiker)	RLB	Y
*Dermatophilus congolensis*	RB	CE	*Theileria spp.* (kudu)	RLB	Y
*Dictyocaulus viviparus* (L1)	FB	7D, 5W, Y	*Theileria spp.* (sable)	RLB	Y
*E.coli*	RB	CE	*Theileria spp.*	MIC, (RLB)	7D, 5W, Y, CE
*Ehrlichia chaffeensis*	RLB	Y	*Theileria taurotragi*	RLB	Y
*Ehrlichia ruminantium*	RLB,MIC,PCR	Y, CE	*Theileria velifera*	RLB	Y
*Ehrlichia spp.* (Omatjenne)	RLB	Y	*Toxocara vitulorum*	FM,FC	7D, 5W, Y
*Eimeria alabamensis*	FM,MIC	7D, 5W, Y	*Trichophyton spp.*	MIC	CE
*Eimeria auburnensis*	FM,MIC	7D, 5W, Y	*Trichostrongylus axei*	FM,FC	7D, 5W, Y
*Eimeria bovis*	FM,MIC	7D, 5W, Y	*Trichuris spp.*	FM,FC	7D, 5W, Y
*Eimeria cylindrica*	FM,MIC	7D, 5W, Y	*Trypanosoma brucei*	HCT,DG,PCR	7D, 5W, Y
*Eimeria ellipsoidalis*	FM,MIC	7D, 5W, Y	*Trypanosoma congolense*	HCT,DG,PCR	7D, 5W, Y
*Eimeria subspherica*	FM,MIC	7D, 5W, Y	*Trypanosoma spp.*	HCT,DG,PCR	7D, 5W, Y
*Eimeria zuernii*	FM,MIC	7D, 5W, Y	*Trypanosoma theileri*	HCT,DG,PCR	7D, 5W, Y
Epizootic haemorrhagic disease	PCR	Y, CE	*Trypanosoma vivax*	HCT,DG,PCR	7D, 5W, Y
*Fasciola spp.*	FS,MIC	7D, 5W, Y	*Weksella zoohelcum*	RB	CE
*Haemonchus placei*	FM,FC	7D, 5W, Y			

*RB* routine bacteriology, *CE* clinical episode, *CL* clinical examination, *RLB* reverse line blot, *7D* recruitment visit, *5W* routine 5 weekly visit, *Y* final visit at 51 weeks, *FM* faecal examination by McMaster’s technique, *FC* faecal culture, *MIC* routine microscopy, *SNP* skin snip and culture, *ZN* Ziehl–Neelsen stain, *DG* dark ground microscopy, *HCT* haematocrit, *PCR* polymerase chain reaction.

In addition, the project screened stored sera from calves at 51 weeks or from their last visit prior to death for evidence of exposure to a number of other diseases believed likely to be important in this region. Further, plasma and DNA were analysed at a number of external laboratories (Table 4).

**Table 4:** Serological screening tests to pathogens

**Pathogen**	**Ab/Ag based**	**Test name**	**Manufacturer**	**Visits tested**
*M. bovis*	Ab	Bovigam ELISA	Prionics	Y
RespiratorySyncitialvirus	Ab	ELISA	Svanova	Y
Bluetonguevirus	Ab	ELISA	PI	Y
*T. parva*	Ab	ELISA	ILRI in house	7D, 5W, Y
*T. mutans*	Ab	ELISA	ILRI in house	7D, 5W, Y
*A. marginale*	Ab	ELISA	ILRI in house	7D, 5W, Y
*B. bigemina*	Ab	ELISA	ILRI in house	7D, 5W, Y
Parainfluenza3 virus	Ab	ELISA	Svanova	Y
Bovine ViralDiarrhoea virus	Ab	ELISA	Svanova	Y
Bovine ViralDiarrhoea virus	Ag	ELISA	Svanova	Y
EpizooticHaemorrhagicdisease virus	Ab	ELISA	PI in house	Y
Akabanediseasevirus	Ab	ELISA	PU in house	Y
Palyam group	Ab	ELISA	PU in house	Y
InfectiousBovineRhinotracheitisvirus	Ab	ELISA	Svanova	Y
*Neospora caninum*	Ab	ELISA	Svanova	Y
*Brucella spp.*	Ab	ELISA	IDEXX	Dam 7D
*Leptospira hardjo*	Ab	ELISA	Linnodee	Dam 7D

PI is the Pirbright Institute (formerly the Institute for Animal Health). *Ab* antibody, *Ag* antigen.

Whole blood samples in EDTA were stored in “magic buffer" (Biogen Diagnostica, Spain) and were genotyped using the Illumina 50K bovine SNP chip (Illumina Inc.®;).

### Database and sample tracking

The project managed data in a set of linked Access databases (Microsoft Corp.). All reports of calf births and recruitment visits were managed in the reporting database. After animals were recruited the main household questionnaire and the routine clinical visits, clinical episodes and post mortems were recorded using palm pilots running Satellite Forms (SatelliteForms.net). These were connected to the field database and daily downloaded. Every animal was tagged with a bar coded ear tag and visit sheets for each individual were kept. At every visit, the bar code was scanned to minimise recording errors. The field database generated a list of samples and then tests that were to be carried out on them in the local Busia laboratory and this was synchronised each evening so the laboratory staff knew what testing to do each day. The laboratory database linked all the barcoded samples in the field database to the respective calf, to the test results, to where the samples and any daughter samples generated from the original field sample were stored and when they were moved to the ILRI lab in Nairobi or to other laboratories outside Kenya. At the end of the field work the field and laboratory databases were merged and moved to a multiuser MySQL database that could be accessed and updated remotely giving all staff access to the data for analysis. All samples eventually were moved to ILRI Nairobi and were appended to the ILRI laboratory information management system for sample management and tracking. Samples where possible were stored in duplicate and only one of the duplicates moved at a time to reduce the risk of losing complete sample sets. At ILRI duplicates are stored in separate buildings in either -20°C or -80°freezers or in vapour phase in large liquid nitrogen biobank chambers as appropriate.

### Tropical Livestock Units

Tropical Livestock Unit (TLU) is a standardising measure used to quantify different types and sizes of livestock. It gives a reference unit that captures the total number of livestock units present in a farm, with 1 TLU being the equivalent to an animal of 250 kg liveweight. One TLU is equivalent to 1 cow, 10 goats or sheep, 5 pigs, 100 chickens, and 0.7 camels [37,38]. This unit has been used for different purposes, including calculating insurable livestock units in the index-based livestock insurance programmes in northern arid areas of Kenya. The different species and sizes of livestock kept in the farms were converted in to TLU’s to serve as a proxy indicator for livestock wealth of each household. The conversion factors used here are those reported by Njuki *et al.*[39].

### Analysis

The R software version 2.9.1 ( http://cran.r-project.org/) was used to generate the descriptive statistics and graphics of the farm characteristics and frequencies of pathogens. All statistical tests were interpreted at the 5% level of significance.

Survival time for each calf was defined as the age at which the study calf died due to infectious causes. Animals that died for reasons other than infectious causes, or that were lost or removed from the study before one year for non-compliance were censored. These contributed “at-risk” time only up to the censoring point. All survivors to one year were censored at the time of leaving the study. Kaplan-Meier estimates of the survival function were used to determine the overall mortality rates [40].

## Results

### Cohort characteristics

**Figure 4:**
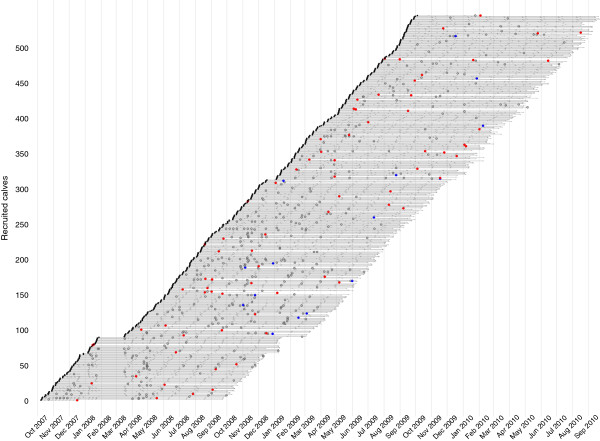
**Life lines for each calf showing the time of recruitment, routine examinations and clinical episodes or deaths over the 3 years of the IDEAL project.** Black dot=recruitment date, grey bar=weekly visit, grey circle=clinical episode; red dot= died and blue dot = euthanised.

A total of 548 calves were recruited and followed for up to 51 weeks or until they died over the 3 year period of the field work. The spatial distribution of the selected sub-locations is given in Figure 1 and the number of calves recruited as a proportion of the breeding dams in each sub-location is given in Table 2. The cattle densities in each sub-location ranged from 220/km^2^ to 2439/km^2^ and the sub-locations ranged in size from 4.38 km^2^ to 22.5 km^2^. The average herd size across all sub-locations ranged from 2.2 breeding cows in Karisa a more hilly area compared to 6.2 animals in Kokare. The life line for each calf is illustrated in Figure 4 and highlights the drop out of calves from death and euthanasia and the pattern of clinical episodes. In addition there were 2 periods where sampling and particularly recruitment were suspended. The first was following the political unrest in 2008 and work in the field was suspended for 6 weeks. This resulted in a small number of calves missing visits for one or two 5 weekly visits. The second was over an extended holiday period in 2009/2010.

### Farm characteristics

**Figure 5:**
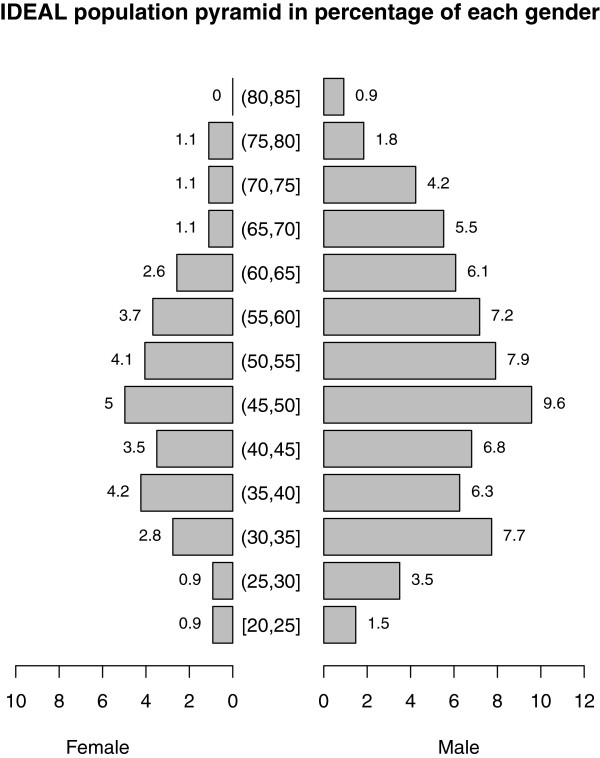
**Population pyramid showing the age structure for male and female household heads.** Each bar value represents the percent number of farmers in that age group.

A total of 548 owners/household heads were interviewed. Data on the owner’s age, gender, education and training level attained, and main occupation are summarised in Figure 5 and table 5. Of the 548 owners, 69% were men and 31% women. The mean age in years for male owners was 50.7 (range 22 - 85) and that for females 49.0 (range 20 - 78). Differences in ages between male and female farmers were statistically insignificant (*p*=0.1679, df= 352, 2-sample t-test). Approximately 15% of the farmers had no formal education, and none had attained university education. A small percentage (21%) had gained technical skills allowing them to work in the informal markets with the common ones being masonry, tailoring and carpentry. The majority (86.2%) of the interviewed owners reported farming as their only source of income, with the rest reporting teaching, civil service, pension and business as their main sources of income with farming offering supplementary income.

**Table 5:** Descriptive statistics for farmer’s demographic variables

	N*	**Frequency**	Percent**
Sex of house head	548		
Male		370	69
Female		178	31
Education level of house head	544		
None		81	14.9
Primary education		337	61.9
Secondary education		126	23.2
University education		0	0
Technical training	541		
No		415	76.7
Yes		126	23.3
Main occupation	544		
Farmer		469	86.2
Teacher		6	1.1
Civil servant		11	2
Business		22	4.1
Retired with pension		14	2.6
Other		22	4

*Not all the farmers responded to the questions in the questionnaires and N notes the number of respondents to the particular question.

**The proportions are calculated using the number of respondents to the question.

The average farm size was only 1.98 ± (0.1 SE) hectares (range 0.1 to 23.1 ha), with majority (96.1%) being owned. Such land is continuously sub-divided, to give adult sons an inheritance and ownership rights. This practice results in families owning small pieces of land that are sometimes not economically viable for agriculture. The rest (3.9%) rented the land they farmed on. All the farms selected for the study kept cattle and also planted food crops, with each farm having a median 5 (range 1 to 131) cattle. The indigenous short horn zebu cattle were the predominant breed kept, with only a small percentage (3.1%) also keeping zebu crosses. Farmers kept more than one species of livestock; an attribute identified as a strategy for spreading risk of losses [41,42]. Different livestock species serve different purposes within the farm enterprise. The general herd structure is given in Table 6, with adult females comprising 41.4% of all cattle kept, and adult males 9.8%.

**Table 6:** Land sizes, livestock species kept and the herd structure

	**N**	**Percent**	**Mean**	**median**	**s.d.**	**Min.**	**Max.**
**Land size owned (hectares)**							
	517	94.3	1.98	1.37	2.28	0.1	23.1
**Livestock numbers**							
All cattle	548	100	6.5	5	7.6	1	131
Indigenous cattle	548	100	6.5	5	7.6	1	131
Cross breds	17	3.1	1.4	1	1	1	5
Goats	209	38.1	3.5	3	3.8	1	33
Sheep	112	20.4	3.9	2.5	5.3	1	48
Pigs	150	27.3	2.2	1	2.2	1	13
Chickens	485	88.5	14.3	10	12.7	1	120
Dogs	297	54.2	2.04	2	1.4	1	9
Tropical livestock units	546	99.6	5.8	4.1	6.71	0.48	114.3
**Herd structure (indigenous)**	548	Frequency	Mean/farm	Percent			
Adult females		1463	2.7	41.4			
Adult males		345	0.6	9.8			
Female calves		465	0.8	13.2			
Male calves		446	0.8	12.6			
Weaning females		399	0.7	11.3			
Weaning males		417	0.8	11.8			
Total		3535	6.5	100.0			

### Husbandry and management practices

Almost 60% of the farms provided housing for livestock. This was usually in the form of an open yard/kraal surrounded by a fence made of untreated wood or bushes with no roof. The remaining 40% of farms provided no housing and the animals were left free or tethered within the homestead during the night. Among those providing housing, 83.1% housed calves separate from the dams/bulls. Calves were not allowed to graze with adults (in 94.4% of the farms) until after weaning. This was mainly to prevent calves suckling dams while out in the field. Calves were allowed to suckle as the farmer milked, with some farmers reporting that milk-let-down in their short horn zebus only happened when stimulated by calves. Other farmers obtained their share first and left the rest for the calf to suckle.

During the dry season, 49.1% of the farms reported providing drinking water for the cattle within the homestead. The rest drove their animals to a water source. These proportions did not differ significantly between the dry and the wet seasons. Distances to the watering points were below 1 km for 73.8% and 75.8% of the farms in the dry and wet seasons respectively, with the rest travelling more than 1 km to access drinking water. Table 7 shows data on the housing, distances to watering points, frequency of watering, and quality of water both in the dry and wet seasons.

**Table 7:** Description of housing, and watering practices in the dry and wet seasons

	**N**	**Dryseason**		**Wet season**	
**Housing**	545	Freq	Percent	Freq	Percent
Kraal/yard		321	58.9	322.0	59.3
None		224	41.1	223.0	40.7
**Access to water**	547				
**Distance to furthest**					
**watering point**					
At homestead		91	16.6	100.0	18.3
<1 km		313	57.2	314.0	57.5
1–5 km		141	25.8	131.0	24
6–10 km		2	0.4	1.0	0.2
**Frequency of**					
**watering**					
Freely available		11	2	13.0	2.4
Once a day		149	27.2	446.0	81.5
Twice a day		367	67.1	87.0	15.9
Thrice a day		20	3.7	1.0	0.2
**Water quality**					
Good, clear		533	97.4	508.0	92.9
Muddy		14	2.6	39.0	7.1

### Cattle trading and breeding practices

Almost all the cattle purchases and sales (98.9%) were done through cattle markets (Table 8). The rest (1.1%) of the farms reported trading animals directly with neighbouring farms. A total of 24 different cattle markets were reported serving the 20 sub-locations, spanning four administrative districts. However, a quarter (6/24) of these markets served 71.2% of all the farmers in the study, an indication that farmers preferred trading in big markets, where they are likely to get more competitive prices.

**Table 8:** Location of trading markets and sources of breeding bulls

	**N**	**Freq**	**Percent**
**Location of**	504		
**purchasing point**			
Within sublocation		75	14.9
Neighbouring sublocation		396	78.6
Other		33	6.5
**Purchasing point**	539		
Market		533	98.9
Neighbouring farm		6	1.1
**Breeding practices**	548		
Own bull (bred)		63	11.5
Own bull (bought)		45	8.2
Bull donated		2	0.4
Bull borrowed		422	77
Communal area bull		19	3.5
Other		1	0.1

There were no reports of organised breeding programmes, and farmers did not keep any written breeding records. The choice of breeding bulls was mostly based on availability of a bull, and if more than one then the farmer decided on personal preferences. Only 11.4% and 8.2% of the farms kept own-bred or purchased breeding bulls respectively (see table 8). Most farmers (76.2%) borrowed breeding bulls whenever their cows needed service. Based on this, only a few bulls are available to serve animals, raising the chances of widespread inbreeding. A few farmers (3.4%) indicated they did not make any direct breeding decisions and depended on their cows being served while grazing in the same communal areas or at watering points. This number is likely to be much higher than reported as animals mix freely and frequently at watering points and communal grazing fields.

### Access to veterinary services

During the farmer interview at the recruitment visit, most farmers (84.7%) reported accessing some form of veterinary services, mainly provided by private animal health workers, and to a lesser extent by government animal health workers, and veterinary drug suppliers (see Table 9). A few farmers indicated they did not use the services of an animal health worker, and instead treated their sick animals themselves. Approximately 90% of farmers reported using some form of tick control of which most (89.9%) reported using whole body spraying with acaricides at the farm. Only a few farmers reported accessing communal cattle dips. Most of the cattle dips in the study sub-locations are abandoned and not in use. Only just over 50% of farmers reported using any form of anthelminthic treatment and only around 18% reported using any form of tsetse control. A moderate proportion of farmers reported using vaccination (52%) although most of those reporting using vaccines did not know what vaccine they had given their animals or what they were protected against (76.7%) and their use seems to be largely driven by need rather than a regular programme of control.

**Table 9:** Description of access to veterinary services and disease control practices in the farm as reported during the calf recruitment visit

		**Frequency**	**Percent**
**Access to veterinary services**	544		
Yes		461	84.7
No		83	15.3
**Type of Veterinary support**	461		
Private animal health worker		264	57.3
Government animal health worker		176	38.2
Veterinary drug supplier		23	5.0
Farmer		10	2.2
**Tick-control**	548		
Yes		498	90.9
No		50	9.1
**Application method**	498		
Spraying whole body		462	92.7
Spraying legs only		9	1.8
Pour on		6	1.2
Hand dressing		25	5.0
Dipping		8	1.6
Other (traditional,manual removal)		10	2
**Worm control**	548		
Yes		309	56.4
No		239	43.6
**Application method**	309		
Drench		265	85.8
Bollet		47	15.2
Others (injectables/unknown)		2	0.6
Traditional		5	1.6
**Trypanosome control**	548		
Yes		98	17.9
No		450	82.1
**Method used**	98		
Spraying whole body		51	52.0
Chemotherapy		32	32.7
Pour-on		10	10.2
Other (dipping/head dressing/			
unknown)		8	8.2
**Use of Vaccines**	546		
Yes		284	52.0
No		262	48.0
**Frequency of use**	277		
Routinely		9	2.9
When need arises		269	97.1
**Vaccine type used**	284		
Unknown		230	81.0
Anthrax		8	2.8
Black quarter		11	3.9
Contagious Bovine Pleural Pneumonia		1	0.4
Foot and mouth disease		25	8.8
Lumpy skin disease		18	6.3
Other		6	2.1

There was a notable difference between the proportion of farmers who reported carrying out disease control measures such as tick control and worming during the initial visit, and the actual proportion of farmers who reported using any preventive measures during the one year follow up period. This suggests that farmers are answering what they think they should be doing or maybe have done but a significant proportion actually then appeared to not carry out these measures over the course of our observations. Interestingly with tsetse control stated and observed activities seem to align well possibly reflecting the recent inputs form NGOs in this area. In contrast vaccination use was much higher than stated and this is not clear why such a discrepancy should arise. This highlights the need for caution in interpreting responses especially from cross-sectional data (see Table 10).

**Table 10:** Table comparing the proportion of farms reporting using each disease control measure at initial visit alongside actual proportion of farms that carried out the measures during the follow up period (n = 548)

**Type of control**	**Initial visit %**	**Actual practice %**
Tick control*		
Yes	90.9	69.9
No	9.1	30.1
Worm control*		
Yes	56.4	26.8
No	43.6	73.2
Tsetse and trypanosome control		
Yes	17.9	14.1
No	82.1	85.9
Vaccine use*		
Yes	52.0	96.4
No	48.0	3.6

*significantly different at the 5% level using a McNemar’s chi-squared test.

### Morbidity and mortality

The 548 recruited calves contributed a total of 175,732 calf days of life to the study. Figure 4 shows the temporal pattern of deaths and clinical episodes over the 3 years of the study. A total of 88 calves died before reaching 51 weeks of age giving an crude mortality rate of 16.4 (13.2-19.5) per 100 calves in their first year of life (Table 11).

**Table 11:** Counts of primary cause of deaths attributed by expert committee

**Cause of death**	**No. calves**
East coast fever	32
Unknown	20
Haemonchosis	9
Heartwater	6
Trauma	3
Actiomyces pyogenes	1
Babesiosis	1
Bacterialpneumonia	1
Black Quarter	1
Cassava	1
Foreign body	1
Mis-mothering	1
Rabies	1
Salmonellosis	1
Trypanosomiasis	1
Turning sickness	1
Viral pneumonia	1
No post mortem carried out	6
Total	88

NB 2 additional calves were considered to have died with ECF as a secondary contributing cause; one with heartwater and another with black quarter.

**Figure 6:**
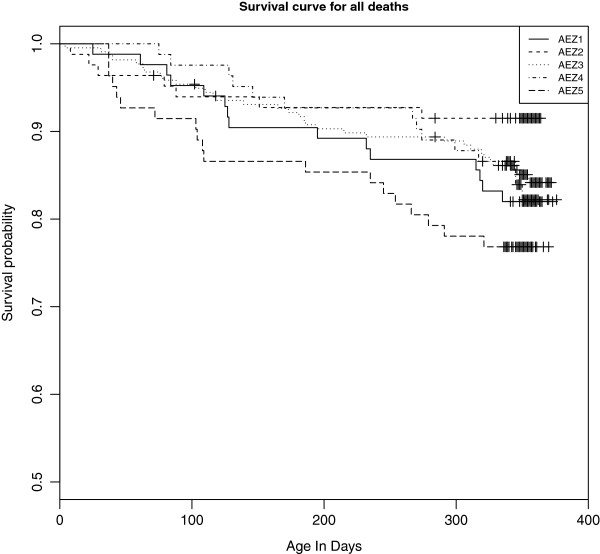
**Kaplan-Meier survival curves for deaths due to all causes.** + mark censoring for reasons other than death and are mainly at 51 weeks when visits stopped. The dashed lines give the 95% confidence intervals on the survival probability.

Fifteen calves were euthanised and were considered to have died from the primary pathology reported on *post mortem*. The distribution of times of deaths by AEZ is given in the Kaplan-Meier plot (Figure 6) showing that AEZ5 which is UM3 in Figure 1 and includes Magombe East, followed by AEZ1 (LM1) which include Bumala A had much higher death rates than other AEZs. The reasons are not yet clear and are the subject of ongoing analyses. Deaths were also attributed to a secondary or contributing cause of death when this was appropriate.

Unfortunately due to logistical reasons *post mortems* were not carried out on 6 of these calves so their cause of death remained unknown. Of the remaining 82 deaths all received a post-mortem examination. A further 4 of this 82 were of unknown cause (a total of 10 calves that died of a completely unknown cause). Seven died from a known non-infectious cause (cassava poisoning (1), foreign body pneumonia (1), mismothering (1), starvation (1), trauma (3)) and 1 died from an unidentified non-infectious cause. Eleven calves had clinical signs indicative of an infectious agent but the definitive cause remained unidentified and 59 died of an infectious cause that was diagnosed by post-mortem examination, appropriate testing and clinical history (East Coast fever (33), turning sickness (1), haemonchosis (10), heartwater (6), babesiosis (1), rabies (1), salmonellosis (1), trypanosomiasis (1), black quarter (1), viral pneumonia (1), multifocal abcessation due to *Actinomyces pyogenes* (1), and *Arcanobacterium* infection (1). This gives a minimum of 70 deaths attributable to infectious diseases and a minimum mortality rate due to infectious causes of 13.3% (10.4-16.2) per 100 calves in the first year of life.

Of the 32 cases of East Coast Fever 8 had a contributing cause of helminthiasis, 5 of which were due to haemonchosis and 2 of trypanosomiasis. Of the 10 cases of haemonchosis 2 had a contributing cause of Theileriosis, and 1 of lung worm (*Dictyocaulus viviparous*). Of the 6 heartwater cases 1 had a contributing cause of East Coast fever as did the case of black quarter. It is interesting to note that in an area generally considered to have high tsetse challenge there seemed to be little clinical trypanosomiasis.

**Figure 7:**
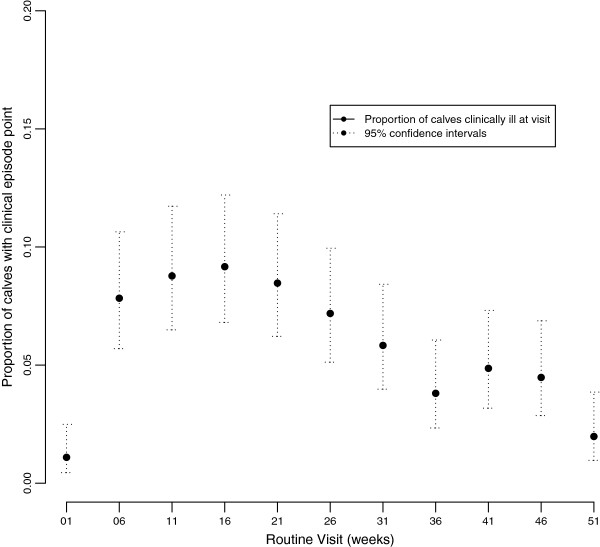
The distribution of the proportion of calves classed as having a clinical episode stratified by visit number over the 51 weeks of observations for each calf in the IDEAL project.

A further 307 clinical episodes were observed by the AHAs on their routine 5 weekly visits and 216 clinical episodes were reported during non routine visits in response to reported illness. The details of all the clinical signs and patterns is currently under analysis but the overall distribution of clinical episodes by age is given in Figure 7. This suggests a bimodal pattern with a large peak around 16 weeks at the time when maternal antibodies might be expected to be waning. There is a second smaller peak later around 41 weeks when many calves are weaned.

### Pathogens and exposures

**Figure 8:**
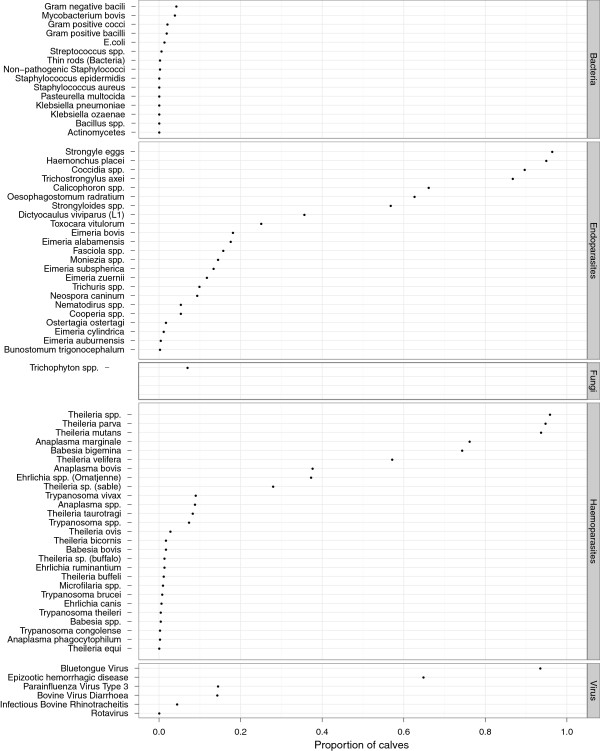
The proportion of animals positive for a given pathogen/test combination at any time through the course of the 51 weeks of observation on each calf in the IDEAL project.

Figure 8 shows the list of pathogen/test combinations experienced by the calf by the time of publication crudely stratified into endoparasites, haemoparasites, bacteria and viruses. Some of the common pathogens such as *Theileria spp.* appear several times as a number of techniques were used to identify them. In addition, some assays, such as microscopy, do not distinguish between species. More detailed analysis of these co-infections is on going. What this Figure shows very clearly is that this population of calves is infected with over 50 different pathogens and has been exposed to at least a further 6 bacteria and viruses. However, relatively few pathogens were found in the majority of calves, and the main pathogens were helminths and protozoan haemoparasites. What is of particular interest is that, given such high incidences of these key pathogens such as *T. parva*, *A. marginale*, *B. bigemina* and *H. placei*, why more of these calves did not die. One of the main objectives of the continuing analyses of this dataset is to unravel the coinfections and relate these to the calf genotype and key outcomes such as growth rate, morbidity and mortality. It is also interesting that there are very few bacterial diagnoses and these appear to have only sporadic occurrence and rarely contributed to death. We plan to look in more detail at the dam serology, but of the 2 bacterial pathogen exposures already measured in the dams, *Brucella spp.* and *Leptospira hardjo* the seroprevalences were extremely low, 0.036 (0.022-0.050 adjusted 95% CI) and 0.068 (0.035-0.101 95% adjusted CI) respectively. Also there was little clinical evidence of some of the major viral diseases such as foot-and-mouth disease.

## Discussion

The IDEAL project is the first attempt to describe the entire disease burden of any naturally occurring population. Funding was only available to follow calves for the first 12 months of life. The use of a longitudinal design, though enormously logistically challenging in this environment, allowed us to generate a unique dataset to study the effects of co-infections in the SHZ breed in this small holder setting. This may be applicable across a large sector of the Great Lakes basin where very similar breeds and husbandry are in operation.

When designing the project a number of different approaches were considered. They included stratification by management system, wealth/herd size, livestock distribution, location, ethnicity, etc. However, the lack of available data on several of these factors led to the decision to stratify by agro-ecological zone only. Random cluster sampling will have ensured that reasonable representation was provided for the various levels of each of the un-stratified factors, i.e. the total sample size will include farmers with varying herd sizes and management systems. The proportion of sub-locations sampled in each AEZ is in proportion of each AEZ in the total survey area (based on numbers of sub-locations). The study was constrained by logistics to an area of 45 km radius from Busia town in order to make repeated visits possible. Initially other options were reviewed but following piloting of sampling in the field it became clear that given the road conditions and number of animals that would have to be sampled per day at the peak of sampling in year 2 this was the most practical approach.

Owners were paid a retainer for the year to allow access to the animals and therefore compliance was very high. There were a small number of instances of animals being stolen and of owners treating the calves with anthelminthics without consulting the project vet. Where these were identified animals were censored and their data from the visit following treatment discarded.

The descriptive analysis from the recruitment interview indicate that livestock production in this system is characterised by low-input, with as few as 30% of the farms carrying out any form of disease control during the follow-up time. Even for those farms that reported carrying out disease control measures, the frequency of these per year was below what would be effective. This level of management would likely be insufficient to support the use of improved "exotic" breeds which are kept in the region but which we intentionally excluded from this study. The Western Province of Kenya accounts for only 4% of Kenya’s total exotic dairy herd [43]. This is despite major breed improvements programs instituted to support smallholder farmers in the region through increased livestock productivity [43,44].

Livestock disease and vector control are required for increased livestock productivity, and prevention of losses through disease-related morbidity, mortality, and loss of markets for livestock products. The observed lack of disease control has implications on some of the strategies envisaged to rapidly improve livestock-dependent livelihoods. It also highlights the need to provide support not just for the imported exotic breeds but also for the indigenous breeds in order to minimise the losses and maximise productivity. The consistent use of disease control practices has contributed to the relative success of the smallholder dairy sector in the Kenyan highlands [45]. The benefits of such controls, carried out at community level, have also been demonstrated in other settings [46]. Failure to consider these disease issues is recognised as a factor that could seriously reduce rural growth [47].

Many countries in sub-Saharan Africa have had to make structural adjustments to their veterinary infrastructure and the services they provide which leaves farmers and herdsmen without the support needed to introduce exotic genetic stock. Further, Rege *et al.*[48] argue that breeding strategies in the context of smallholder farms should be based on improving food security, income and overall livelihoods of the livestock keepers and should not be based on genetic improvement of livestock. Focus should be on providing the most appropriate genotypes in a local context. However, identifying these appropriate genotypes is itself complex. Mwachara *et al.*[49] identify the need to involve the livestock keepers in designing the breeding programmes to take into account the full array of contributions to livelihoods that these animals make and so identify genetic characteristics related to these functions. Whereas most programs have concentrated on cross-breeding, there exists a lot of potential and advantages for improvements based on within-breed selection.

The mortality rates in this indigenous calf population were higher than anticipated at the design stage. There are few reports that we could find from similar systems but other reports from the region suggest a range of mortalities. Barnett [50] reported a mortality rate of 29% from a study based in Western Province Kenya. In a Tanzanian smallholder dairy system mortality rates of 35% were reported [51] within the first year with 42% reported as of unknown cause and 19% due to redwater (babesiosis). Swai *et al.*[52] reported mortality rates of 12% in small holder dairy systems in Zimbabwe with 56% ascribed to tick borne disease particularly east coast fever. Gitau *et al.*[53] reported 7% mortality in calves up to 6 months of age from the same area of Western. A more recent large study of calf mortality in Mali [54] reported an overall calf mortality of 17% but when this was broken down by system the more intensive systems had high mortality rates of 19% and 25% compared to 10% in the traditional pastoralist systems. Interestingly they report gastrointestinal disorders as causing 28% of their overall mortality followed by perinatal problems (16%) and accidents (14%). Direct comparisons are very difficult to make with many of these studies as the design, breeds, environment etc are not the same. However, it is useful to get an overall impression of how these animals are performing in this system. The mortality rate in the IDEAL cohort appears high given it is an indigenous breed that might be expected to have had time to adapt to the conditions. There are likely to be many contributing causes including possible inexperience in raising cattle compared to traditional cattle owning groups such as the Maasai or Fulani and the co-infection combinations present in the region.

The identification of pathogens at all time points in the study is on going. We adopted a very pragmatic approach using the best field techniques available as the method of diagnosis but for many pathogens this is not sufficient. For example speciation of *Theileria* parasites requires more detailed analysis such as RLB [55]. It must be noted that detection of pathogens is limited by the sensitivity of the assay, the presence of the pathogen at the time of sampling and its location in the tissue which is sampled. This presents many challenges in trying to produce a definitive list of pathogens at every time point for each calf. For this preliminary presentation of the pathogens we have simply summed across all visits to estimate the proportion of calves with each pathogen (or pathogen/test combination). This ignores the dynamics of the order of exposure but this is to be reported in a number of other papers. The list of pathogens is extensive but there are actually only a few very high prevalence pathogens. Theses are mainly gut helminths and tick borne haemoparasites, in particular *T. parva*.

The IDEAL project provides unique data on total livestock disease burden in the region, which will allow for ranking of infectious diseases in order of importance. Such data are important for prioritising interventions, the absence of which up to this point has led to a lack of metrics to assess the impact of livestock diseases leads and therefore inefficient resource allocation [2]. In addition, the project will provide data on the within breed variation of key traits such as growth rates, clinical tolerance and resistance, and survival. This provides a basis for identifying desirable traits that may be taken up while designing within-breed improvement programs. Within-breed selection may not achieve increased productivity per animal as rapidly as cross-breeding methods, but offers the opportunity to retain the adaptive characteristics already present in indigenous breeds and which may offer opportunity for adaptation to changing climates. The findings of positive associations between knowledge of diseases and access to veterinary support with whether farmers carry out disease control practices supports the idea that increased extension services would have significant positive effect on livestock productivity.

## Conclusions

This population of calves is the first to have a comprehensive investigation of the pathogen burden and exposures of any animal population. The analyses of the biobanked samples will continue and it is expected that there will be further pathogens added to the list. This preliminary report on the IDEAL project design and data collection offers an overview of the farming system in Western, Kenya, and of the infectious disease challenges experienced by the calves of the region. It provides a detailed description of the methods used to collect this detailed longitudinal dataset. This provides more information for those reading analytical papers from the project, and acts as a supporting document to the extensive biobank held at ILRI, Nairobi. It gives preliminary results and offers an overview of more detailed analyses that result from the IDEAL project.

## Competing interests

We declare that non of the authors at the time of the study or preparation of the paper have any competing interests that could influence or bias the content of this paper.

## Authors’ contributions

MW conceived the original idea and design and directed the project; MB was responsible for the study design, development of the clinical tools such as questionnaires, sample flows and diseases to be screened, data management and analysis and training of field staff; JP was responsible for the design and random sampling protocols, database management and data analysis; IH was responsible for management of the database, data extraction and analysis; HK was responsible for parasitology protocols and overall managing the project, the sample flows and diseases to be screened for and biobank management; PT was responsible for the management and interpretation of the serological screening of samples; KC was responsible for the clinical protocols, postmortem protocols, interpretation of clinical and postmortem data and training of the field staff; and OH and MN were responsible for managing the phenyotypic protocols and analysis and interpretation of the genetic data components. ST, IC and AJ were the veterinary surgeons in the field and as well as carrying out the clinical work and managing staff on the ground participated in the development of the tools and interpretation of the results. OT designed and implemented the database for the project and contributed to the design of the study. All authors read and approved the final manuscript.
